# New Advances in Dye Analyses: In Situ Gel-Supported Liquid Extraction from Paint Layers and Textiles for SERS and HPLC-MS/MS Identification

**DOI:** 10.3390/molecules28145290

**Published:** 2023-07-08

**Authors:** Adele Bosi, Greta Peruzzi, Alessandro Ciccola, Ilaria Serafini, Flaminia Vincenti, Camilla Montesano, Paolo Postorino, Manuel Sergi, Gabriele Favero, Roberta Curini

**Affiliations:** 1 Department of Chemistry, Sapienza University of Rome, P. le Aldo Moro 5, 00185 Rome, Italy; adele.bosi@uniroma1.it (A.B.);; 2 Department of Earth Sciences, Sapienza University of Rome, P. le Aldo Moro 5, 00185 Rome, Italy; 3 Institute for Complex System, National Research Council, Sapienza University, Piazzale Aldo Moro 5, 00185 Rome, Italy; peruzzi.1957090@studenti.uniroma1.it; 4 Department of Physics, Sapienza University of Rome, P. le Aldo Moro 5, 00185 Rome, Italy; 5 Department of Environmental Biology, Sapienza University of Rome, P. le Aldo Moro 5, 00185 Rome, Italy

**Keywords:** dyes, in situ extraction, hydrogels, SERS, dLLME, HPLC-MS/MS, colorimetry

## Abstract

To date, it is still not possible to obtain exhaustive information about organic materials in cultural heritage without sampling. Nonetheless, when studying unique objects with invaluable artistic or historical significance, preserving their integrity is a priority. In particular, organic dye identification is of significant interest for history and conservation research, but it is still hindered by analytes’ low concentration and poor fastness. In this work, a minimally invasive approach for dye identification is presented. The procedure is designed to accompany noninvasive analyses of inorganic substances for comprehensive studies of complex cultural heritage matrices, in compliance with their soundness. Liquid extraction of madder, turmeric, and indigo dyes was performed directly from paint layers and textiles. The extraction was supported by hydrogels, which themselves can undergo multitechnique analyses in the place of samples. After extraction, Ag colloid pastes were applied on the gels for SERS analyses, allowing for the identification of the three dyes. For the HPLC-MS/MS analyses, re-extraction of the dyes was followed by a clean-up step that was successfully applied on madder and turmeric. The colour change perceptivity after extraction was measured with colorimetry. The results showed ΔE values mostly below the upper limit of rigorous colour change, confirming the gentleness of the procedure.

## 1. Introduction

While sample reduction and procedure miniaturisation are generally desirable in analytical chemistry, the principle of minimal invasiveness is imperative when analysing cultural heritage and, where possible, completely noninvasive analyses are preferred.

Nonetheless, when sampling is forbidden the study of organic components is disadvantaged. Dyes, in particular, represent one of the most complex challenges because of their low concentration and tendency to fade. When they are in complex matrices, care must be taken not to completely lose information about their presence. Noninvasive techniques, like fibre optic reflectance spectroscopy (FORS) or fluorescence spectroscopy, were proved to be effective, in some instances, for dye identification in rather simple matrices [1,2,3,4,5], but they generally lack specificity. Because of their strong fluorescence, dyes are not suited for Raman spectroscopy identification. However, fluorescence can be quenched when signals are enhanced by means of metal nanoparticles (NPs), and very good dye spectra can be obtained by means of surface enhanced Raman spectroscopy (SERS) [6,7,8,9,10]. Nonetheless, the phenomenon occurs when the analyte interacts with metal NPs, which cannot be applied directly on the artifact and, thus, micro sampling is required anyways [6,11]. To overcome this problem, different kinds of solid substrates have been proposed over the last decade. For instance, cellulose films incorporated with NPs can be applied directly on the artifact’s surface and, following SERS measurements, can be removed, therefore limiting the residues left behind [12].

Gels, on the other hand, can be loaded with extraction solvents and used to perform extractions directly from the artifacts. Gels can be prepared with NPs inside or covered with NPs after removal from the artifact, just before undergoing SERS analysis in the place of samples [13,14,15,16,17,18]. Hydrogels, in particular, because of their water retention and release properties, are appreciated for in situ extractions by means of aqueous solutions. Agar gel prepared with Ag NPs has repeatedly been used to obtain a Ag-agar gel support for SERS analyses [14,15,16,17,18]. A Ag-agar gel loaded with an extraction solution was applied on an artifact surface for dye extraction and then left to dry for SERS analyses. While drying, an agar gel shrinks consistently, promoting the close interaction of Ag colloids with the dyes, thus enhancing the SERS signals [19].

**Figure 1:**
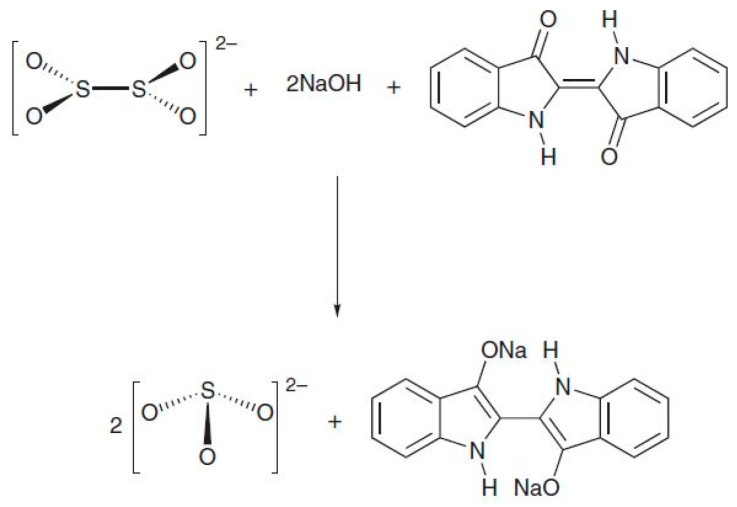
Indigo reduction reaction into leucoindigo using sodium hydrosulphite in alkaline solution: sodium hydrosulphite is oxidised into sodium sulphite (S^III^ → S^IV^), releasing two electrons [28].

However, in spite of SERS efficacy, when it comes to dye analyses, it must be said that high-performance liquid chromatography (HPLC) coupled with diode array detector (DAD) and mass spectrometry (MS) specificity is unrivalled. Hence, when possible, the common procedure involves removing a paint sample larger than 200 µm, or 1–2 mm of thread [6], for the dye extraction from the matrix. But complications with dyes are yet to end; extraction is a very critical and debated step, and useful information can be lost even with a consistent sample in hand. This is because traditional extraction methods imply the use of HCl and high temperatures. These harsh conditions are needed to break the chemical linkage between dyes and their metal substrate. Nonetheless, this way the linkage between glycosylated dyes and their sugar moiety becomes broken, too. Even if dye identification is possible based exclusively on aglycones, glycosylated molecules can be extremely precious to obtain specific information [20]. Taking this into account, several alternatives to HCl have been proposed [21,22,23,24,25,26], from mild acids to organic solvents, mostly supported by high temperatures. In 2015, a completely new basic approach, based on ammonia, was proposed for anthraquinone dyes [27]. The extraction solution, composed of NH_3_, Na_2_EDTA, and NaCl, is able to break the linkage between dyes and their metal substrates, preserving the entire glycosylated molecules at ambient temperature. The resultant analytical composition of ammonia extracts matches the source composition reported in the literature [27]. In 2020, the ammonia–EDTA extraction method was combined with hydrogel-supported microextraction in an attempt to coalesce SERS with the HPLC-MS/MS identification of dyes for the first time [19]. This procedure was designed to obtain as much information as possible on the analytes while preserving their molecular pattern together with the artifact’s soundness. The ammonia solution, being aqueous, is very suited to hydrogel extraction, and its ability to work at ambient temperature makes direct application on the object possible. After extraction from wool, anthraquinone dyes were re-extracted from the gel support, and an additional liquid–liquid extraction was performed to purify the analytes and eliminate Na_2_EDTA and NaCl, both of which are not compatible with the HPLC-MS/MS system. To test the procedure, agar gel and Nanorestore Gel^®^ HWR (high water retention), designed and produced by the Italian Center for Colloids and Surface Science (CSGI), were compared. The outcomes revealed that while SERS analyses provided good results, traditional liquid–liquid extraction was not able to recover the small amount of analyte extracted, and a dispersive liquid–liquid microextraction (dLLME) clean-up procedure was thus developed and validated for this purpose. The development and validation study, which comprehends the application of this methodology to archaeological textiles, is under submission to another journal by the same authors. During the presented work, the whole procedure was also adapted to paint layers and extended to additional dye classes. Three textile mock-ups were prepared dying wool using madder, indigo, and a direct dye: turmeric. Furthermore, madder lake pigment and indigo in powder were mixed with egg yolk to obtain tempera paint mock-ups. While the ammonia–EDTA extraction and dLLME clean-up procedure was used on madder and turmeric, indigo gels were imbibed into an aqueous solution of NaOH:Na_2_S_2_O_4_ 1:2, able to reduce indigotin into its water-soluble form, leuco indigo [16,28] (Figure 1). Once in the hydrogel pores, leuco indigo re-oxides into indigotin when in contact with the atmosphere. A dLLME clean-up procedure was then tested to re-extract indigoids from the gels for the HPLC-MS/MS analyses. All details are listed in Section 3 (Materials and Methods).

## 2. Results and Discussion

### 2.1. Extraction

#### 2.1.1. Madder and Turmeric

**Figure 2:**
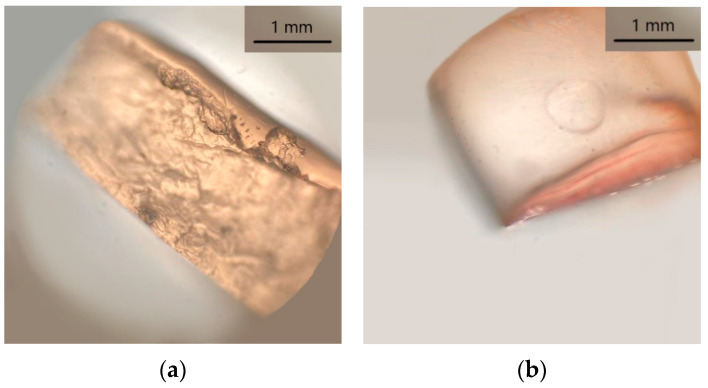
(**a**) Agar gel (3% *w*/*v*) after 3 h extraction from wool dyed with madder; (**b**) Nanorestore Gel^®^ HWR after 3 h extraction from wool dyed with madder.

The ammonia–EDTA extraction solution was visibly able to extract madder and turmeric dyes both from wool and paint layers. As already observed by Germinario et al. [19], agar gel was homogeneously coloured after extraction (Figure 2a), while for the Nanorestore Gel^®^ HWR, the dyes appeared more concentrated on the contact surface (Figure 2b), in accordance with its high retention power.

#### 2.1.2. Indigo

**Figure 3:**
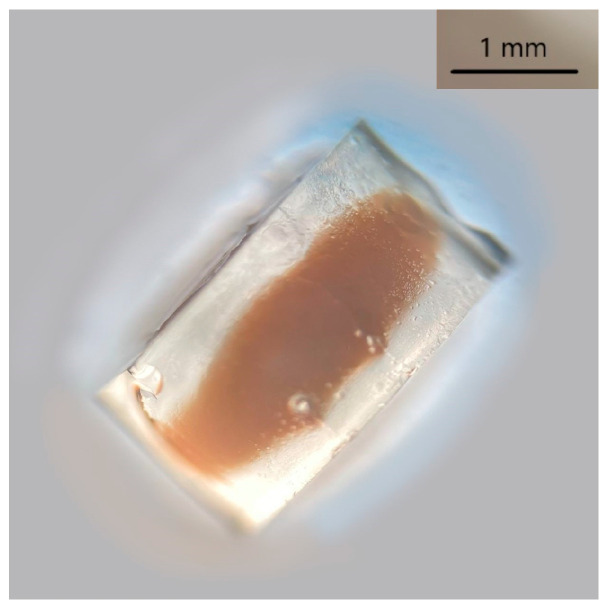
Nanorestore Gel^®^ HWR after 90 min soaking in aqueous solution containing NaOH:Na_2_S_2_O_4_ 1:2.

Agar gel was extremely effective for indigoid dye extraction from both wool and paint mock-ups. The gel appeared greenish coloured right after extraction; however, after contact with the atmosphere, the oxidation of leucoindigo back to indigotin slowly turned the colour back to blue. Conversely, the Nanorestore Gel^®^ HWR showed not to be compatible with the reducing solution. After soaking, the gel appeared altered by look and by touch. A substance absorbed from the solution or degradation product was observable right at the centre of the gel cylinder (Figure 3), and the Nanorestore Gel^®^ HWR consistency was hardened.

### 2.2. SERS

#### 2.2.1. Madder and Turmeric

**Figure 4:**
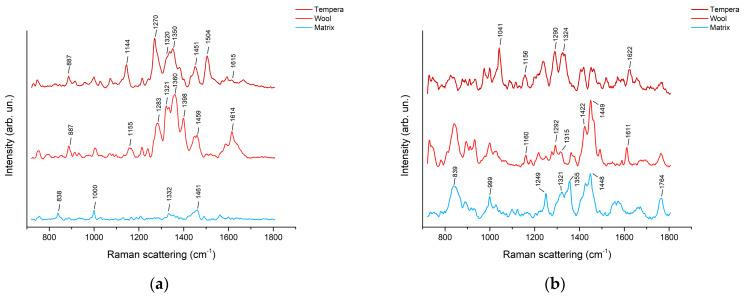
(**a**) SERS spectra of agar gel (3% *w*/*v*) after 15 min extraction of madder from tempera paint and 3 h extraction of madder from wool; (**b**) SERS spectra of Nanorestore Gel^®^ HWR cylinders after 15 min extraction of madder from tempera paint and 3 h extraction of madder from wool.

Spectra were acquired directly on the gels after the addition of Ag colloids. The colloids were made in pastes to prevent excessive absorption into the gel cylinders (see Section 3 (Materials and Methods)). The spectra recorded show SERS scattering peaks attributable to madder and turmeric dyes (Table 1). Peaks at approximately 1270–1280 and 1320 cm^−1^, observed in the spectra of the agar gel after madder extraction from textile and paint mock-ups (Figure 4a), are diagnostic for the presence of alizarin and are attributed to the C-C stretching, H-C-C, and C-C-C bending modes of the anthraquinone ring [15,16,17,18,19,29,30,31,32,33,34].

The peak at approximately 1615 cm^−1^, mainly visible in the spectrum from wool extraction, is attributed to the C=O stretching of the anthraquinone ring [14,15,29,30]. The peak at approximately 1150 cm^−1^ (1144 for tempera and 1155 cm^−1^ for wool) can be ascribed to the C-C stretching and C-H bending modes of the dye molecules [29]. Additional medium and weak intensity peaks between 800 and 1000 cm^−1^, such as the one at 887 cm^−1^, are attributed mainly to anthraquinone skeletal vibration [14,15,29]. The peaks at approximately 1450 cm^−1^ are generally attributed to alizarin C-O stretching and C-O-H and C-H bending [14,15,30]. The scattering band at 1398 cm^−1^, on the other hand, is diagnostic for the presence of purpurin [30,34,35]. This peak is reproducibly more pronounced after extraction from wool than from tempera and could be due to the different preparation recipes. Additional peaks, such as the ones at approximately 1360 and 1505 cm^−1^, could not be assigned to specific modes of anthraquinone dyes and can, hence, be related to the complex molecular pattern extractable from madder roots. Scattering peaks at approximately 1330–1350 cm^−1^, however, were already observed in the agar gel [19,35], and its attribution, hence, remains uncertain.

Madder spectra acquired on Nanorestore Gel^®^ HWR (Figure 4b) similarly exhibit peaks at approximately 1155, 1290, 1320, and 1620 cm^−1^. The peaks were less intense in comparison to the ones from the gel matrix, especially after extraction from wool. In this latter case, peaks at approximately 1422 and 1449 cm^−1^, with a shoulder at 1465 cm^−1^, were preferentially enhanced. These peaks are generally attributed to alizarin C-O stretching and C-O-H and C-H bending [14,15,29]. Nonetheless, peaks in the same positions were also observed in the gel matrix. In addition, the Nanorestore Gel^®^ HWR matrix presents some peaks at approximately 1320–1350 cm^−1^ that overlap with the ones typical of alizarin. A peak at 1041 cm^−1^, visible after extraction from tempera, is attributed in the literature to the alizarin ring C-C-C in-plane bending [29]. The intensity of this specific peak in this spectrum could be due to the geometry of the interaction between the analytes and the AgNPs.

In general, on the paint layers as on the textile, the typical anthraquinone dye SERS scattering peaks were more recognizable after agar-gel-supported extraction. This is in accordance with already published results [19]. This can be related to the high water retention of Nanorestore Gel^®^ HWR, which could, in some instances, hinder the extraction solution release and, hence, the analytes collection.

**Figure 5:**
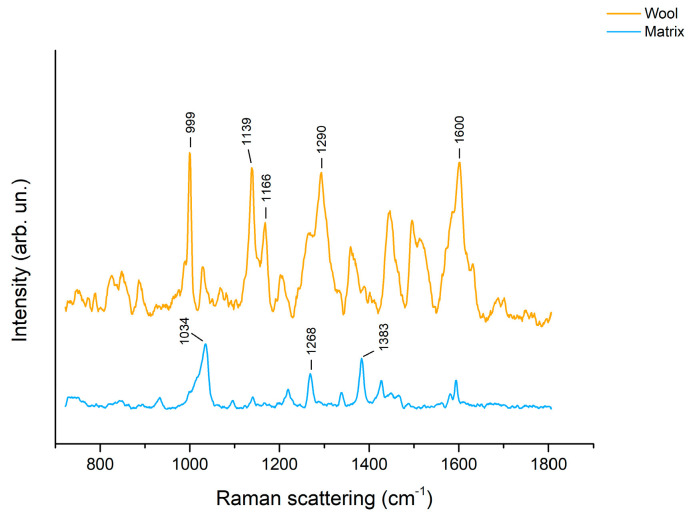
SERS spectra of agar gel (3% *w*/*v*) after 3 h extraction of turmeric from wool.

In the case of turmeric, too, typical SERS scattering peaks could be observed on the agar gel after extraction from wool (Figure 5). Signals at 1139 and 1166 cm^−1^ can be assigned to the molecular skeleton vibration, while the signal at 1290 cm^−1^ is due to the phenolic ring C-C-C, C-C-H, and C=CH bending [36]. Lastly, the band at 1600 cm^−1^ can be assigned to the C-OH bending and its shoulder at 1630 cm^−1^ to the C=C and C=O stretching [36,37]. The peak at 999 cm^−1^ is likely due to the gel matrix. On the contrary, no peaks related to curcumin could be observed on Nanorestore Gel^®^ HWR after the dye extraction. As mentioned for madder, the difference in the extraction capabilities can be explained by taking into account the difference in water retention.

In general, the gel matrices exhibited rather reproducible signals. While the agar gel peak positions are reproducible, anyways, their intensities can change consistently. This fact is a consequence of the agar gel macromolecular structure, which can have variable interaction with AgNPs.

The signals observed on the Nanorestore Gel^®^ HWR blank were also quite reproducible during the experiments that were carried out, and the main scattering peaks were at 839 cm^−1^, 999 cm^−1^, 1355 cm^−1^, 1422, and 1448 cm^−1^.

To avoid any attribution uncertainty, however, especially in the region around 1320 and 1450 cm^−1^, SERS analyses of dyes re-extracted from the gels will be attempted in the future.

#### 2.2.2. Indigo

Because of the degradation that occurred in the reducing solution, the Nanorestore Gel^®^ HWR was not able to extract indigo. Spectra were, thus, acquired only on the agar gel after indigo extraction and Ag colloid pastes addition.

**Figure 6:**
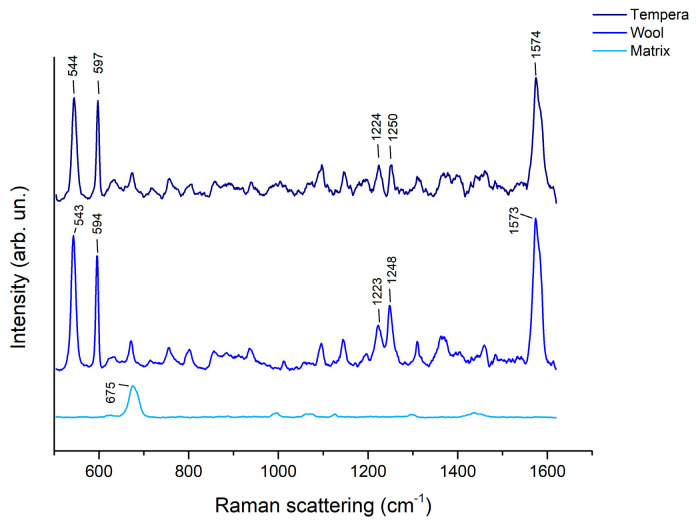
Spectra of agar gel (3% *w*/*v*) cylinders after 5 min extraction of indigo from tempera and from wool.

In the spectra recorded, intense and sharp peaks related to indigoids were visible (Figure 6 and Table 2).

The intense peaks at 544 and 597 cm^−1^ are related to the indigotin C=C-CO-C and C-N bending modes and to the C-C and C-N bending modes, respectively. The peak at 1224 cm^−1^ is due to the molecule N-H and C-H in-plane bending, while the one at 1250 cm^−1^ is due to the C-H, C=C, and N-H in-plane bending [16]. The strong signal at 1574 cm^−1^ can be attributed to the molecule’s C=C and C=O stretching modes [16,38].

The SERS peaks obtained are consistent with the ones published by Platania et al. [16], who extracted indigo from cotton using agar gel loaded with the same reducing solution (NaOH:Na_2_S_2_O_4_ 1:2). Nonetheless, in comparison to the spectrum obtained by Platania et al., the spectra reported here exhibit a pronounced enhancement of the peaks at 545, 597, and 1573 cm^−1^. Interestingly, the spectra obtained after extraction from wool and after extraction from tempera are very similar in spite of the different preparation recipes and are highly reproducible.

### 2.3. HPLC-MS/MS

#### 2.3.1. Madder and Turmeric

**Figure 7:**
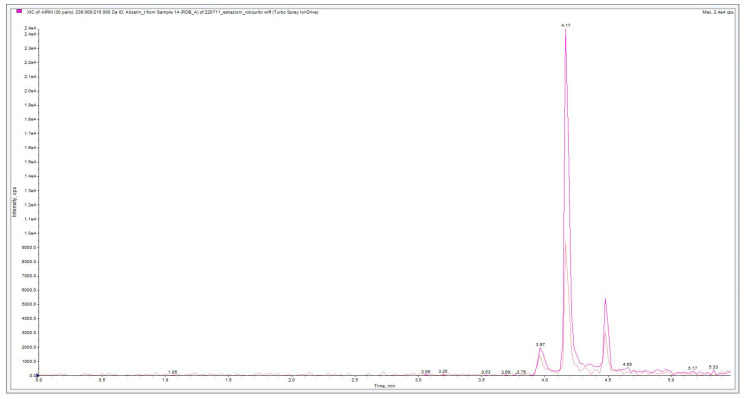
Alizarin chromatogram relative to agar gel (3% *w*/*v*) madder extraction from wool. The chromatogram in darker pink refers to the transition from 239 to 210 *m*/*z*, while the chromatogram in lighter pink refers to the transition from 239 to 167 *m*/*z*.

**Figure 8:**
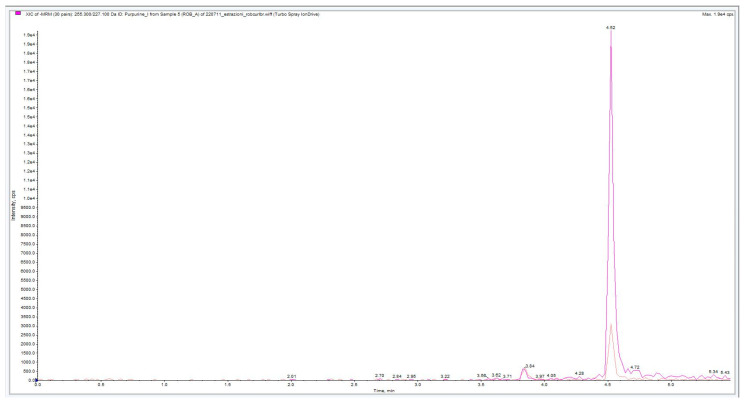
Purpurin chromatogram relative to agar gel (3% *w*/*v*) madder extraction from wool. The darker chromatogram refers to the transition from 255 to 227 *m*/*z*, while the lighter chromatogram refers to the transition from 255 to 171 *m*/*z*.

The chromatograms obtained show the presence of the target analytes selected. The chromatographic peaks related to alizarin fragmentation (Rt = 4.17 min) and purpurin fragmentation (Rt = 4.52) could be observed after re-extraction from agar gel applied on wool (Figure 7 and Figure 8).

On the contrary, re-extraction from Nanorestore Gel^®^ did not produce satisfying results.

The chromatographic peak areas of the analytes are greater for agar gel (3% *w*/*v*) extraction than for Nanorestore Gel^®^ HWR extraction. Again, this is in accordance with their different water retention.

Regarding the extraction from tempera paint layers, no peaks related to alizarin and purpurin were observed. Nonetheless, considering the SERS results obtained, the extraction times from the paint mock-ups could be adjusted to further increase the amount of analyte collected.

The dLLME clean-up procedure, successfully tested and validated for aglycones and glycosylated dyes, demonstrated efficacy also with turmeric. The chromatographic peaks related to curcumin transitions (Rt = 4.68) could be observed after re-extraction from agar gel and Nanorestore Gel^®^ HWR. No peaks related to the analyte were observed in the reference blanks.

#### 2.3.2. Indigo

Because of the extraction solution complexity, the clean-up procedure developed for indigoids (see Section 3 (Materials and Methods)) could not be perfected. The formation of a sulphur salt was observed both in the aqueous phase and organic phase after dLLME. For this reason, HPLC-MS/MS analyses of indigoid dyes with this procedure requires further studies, and the protocol is still under refinement. Considering the good results obtained by means of SERS, however, the reducing solution could be further diluted to hinder the salt precipitation.

### 2.4. Colorimetry

#### 2.4.1. Madder and Turmeric

The colour variations induced using gel-supported liquid extraction are mainly around the colour tolerance applied in the industrial field (ΔE = 3 CIELAB units) considered the upper limit of rigorous colour tolerance (Table 3) [39].

On madder, only Nanorestore Gel^®^ HWR extraction from tempera paint induced a colour variation higher than three. However, no mark was visible on the paint to the authors’ eyes. Agar gel, on the other hand, induced a colour variation of 2.97 CIELAB units, and showed better results when analysed by means of SERS. On wool, both Nanorestore Gel^®^ HWR and agar gel extraction was invisible, leaving a large margin for further developments.

Turmeric colour measurements showed that the dye is sensitive to the extraction solution. After 3 h, a light shift to a reddish colour was effectively visible under careful observation. Nevertheless, the presence of turmeric could be detected using both SERS and HPLC-MS/MS, which means there could be a margin for a reduction in the extraction time.

#### 2.4.2. Indigo

The reducing solution proved to be harsher than the ammonia–EDTA solution. The extraction time was reduced from 3 h to 5 min on wool, providing very good SERS results and a colour variation of only 1.52 CIELAB units (Table 3). Conversely, the extraction on tempera induced a colour variation of 4.14 CIELAB after 5 min. Considering the spectra obtained, the reducing solution could be further diluted and the extraction times adjusted.

## 3. Materials and Methods

### 3.1. Materials

Madder roots (*Rubia tinctorum* L.) and alum (KAl(SO_4_)_2_) were purchased from “Chroma” srl. Genuine indigo in pieces (Indigofera Tinctoria), rabbit skin glue, and CaSO_4_ were purchased from Kremer Pigmente (Germany). Bricks were purchased from “Mattone Romano” srl (Italy). KC_4_H_5_O_6_ was purchased from a grocery shop. Turmeric was purchased from Chroma. Agar in powder (ash 2.0–2.4%), solvents, and salts, such as ammonia (30–33%), NaCO_3_ (≥99.0%), Na_2_S_2_O_4_ (≥82.5%), K_2_CO_3_ (with impurities ≤55.0 ppm), NaOH (≥95%), hydroxylamine hydrochloride (99.9%), NaCl (with impurities ≤0.005% as insoluble matter), Na_2_EDTA (with impurities ≤0.005% as insoluble matter), silver nitrate (≥99.0%), HCl (37%), HCOOH (≥95%), 2-propanol, and 1-pentanol, were purchased from Sigma-Aldrich. Nanorestore Gel^®^ High Water Retention was purchased from CSGI (Center for Colloid and Surface Science).

### 3.2. Lake Pigments Preparation

Red madder lake pigment was prepared following a recipe from Daniels et al. [40]. Briefly, 5 g of madder roots were soaked in 150 mL distilled water and left overnight. The roots in water were heated up until 70 °C for 30 min. After filtration, 2.5 g potassium alum was added to the solution, and the temperature was brought to 80 °C. Meanwhile, 0.94 g K_2_CO_3_ was dissolved in 25 mL water and gently poured into the dye bath under continuous stirring. The lake pigment formed was left to precipitate overnight. Once precipitated, the lake pigment was filtered and finely ground.

### 3.3. Paint Mock-Ups Preparation

Paint mock-ups were made on bricks prepared with eight layers of CaSO_4_ and rabbit skin glue and applied with a brush [41]. The organic pigments were mixed with a solution of 2 mL water and an egg yolk. The pigments were mixed with the binder in a rough proportion 2:1 (*w*/*v*) using a spatula and then applied by means of a brush, and then left to age naturally for six months.

### 3.4. Wool Dyeing

Textile mock-ups were prepared from wool dyed using madder, indigo, and turmeric. First, 2 g wool yarn was mordanted as follows: 620 mg alum and 120 mg potassium bitartrate (KC_4_H_5_O_6_) were dissolved in 250 mL of distilled water. The solution was warmed to 40 °C and kept for ten minutes. Wool was soaked in the solution once cooled down. The temperature was increased again to 80 °C for 40 min and kept for 1 h under gentle magnetic stirring [27]. Once at ambient T, the wool was removed, squeezed, and left to dry.

To dye wool using madder, 1 g madder roots was soaked in 400 mL distilled water and warmed to 35 °C. One gram of mordanted wool was soaked, the temperature was brought to 80 °C over 40 min, and then kept for 1 h under gentle magnetic stirring [27].

For direct wool dyeing using turmeric, 0.5 g turmeric in powder was soaked in 400 mL distilled water, brought to 90 °C, and then kept for 15 min under magnetic stirring. Successively, 1 g unmordanted wool was soaked and left for 30 min at the same temperature.

To dye wool with indigo, 0.6 g ground indigo was added to 10 mL distilled water at 45 °C. Successively, a solution of 0.6 g NaCO_3_ in 6 mL distilled water and a solution of 1.5 g Na_2_S_2_O_4_ in 50 mL distilled water at 45 °C were added one after the other. The mixture obtained was brought to 55 °C and kept for 20 min. Afterwards, 3 g unmordanted wool was soaked in the bath and left for 10 min. The wool was then removed and left in contact with the atmosphere to allow the dye to re-oxidise.

After the dyeing was performed, distilled water was always used to wash the wool until the rinse was transparent; then, it was left to dry and age naturally for six months. The textile mock-ups were made by winding dyed wool yarns around microscope glass slides.

### 3.5. Agar Gel Preparation

For the agar gel preparation, 0.24 g agar in powder was added to 8 mL distilled water in a 100 mL beaker. The beaker was then gently shaken in a boiling water bath for 10 min so that the polymer reached its melting temperature. The solution was cooled for 30 min at ambient temperature and stored in a refrigerator overnight [19,35].

### 3.6. Ag Colloids Pastes Preparation

The Ag colloids were prepared following the protocol developed by Leopold and Lendl [19,42]. Specifically, 20 mg NaOH was added to 5 mL MilliQ water, and 21 mg NH_2_OH · HCl was added to another 5 mL MilliQ water. The two solutions were mixed together and poured into a solution of 17 mg AgNO_3_ in 90 mL MilliQ water under constant magnetic stirring. The obtained Ag colloid solution was stored in the fridge. To obtain colloidal pastes, 10 mL colloids were centrifuged at 4500 RPM for 20 min, and the supernatant was discarded [43,44].

### 3.7. Gel-Supported Liquid Extraction

**Figure 9:**
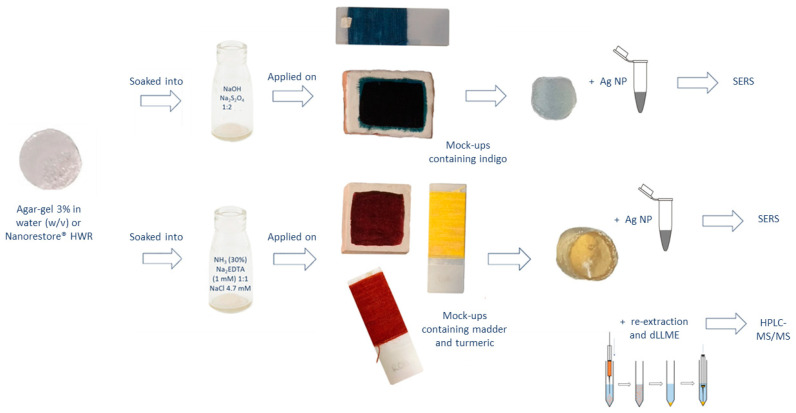
Schematic diagram of the whole gel-supported liquid extraction workflow for SERS and HPLC-MS/MS identification.

Agar gel 3% (*w*/*v*) and Nanorestore Gel^®^ HWR, as a commercial product, were cut into cylinders of approximately 4 mm in diameter. The cylinders were soaked for 90 min in a solution of NH_3_ (30–33%) and Na_2_EDTA 1 mM (1:1). NaCl was added until a final concentration of 4.7 mM [19,20,27]. After 90 min, the gel cylinders were removed using tweezers and left to lose 5% of their weight. In the case of indigo extraction, after soaking in the reducing solution, the gel cylinders were quickly dried on absorbent paper and applied directly on the mock-ups to avoid prolongated contact with O_2_ in the atmosphere. The gel cylinders were applied on paint and textiles with glass slides on the top to prevent solution’s evaporation. The extraction time varied, from 5 min on the paint layers to 3 h on the textiles. The whole procedure workflow is summarised in Figure 9 as a schematic diagram.

### 3.8. SERS Analysis

For the SERS analyses, 20 µL of colloidal pastes were poured on the gel face, which was in contact with the mock-ups. The gels were then left to dry for 12 h at ambient temperature. The SERS analyses were carried out directly on the dry gel using a Horiba Jobin-Yvon HR Evolution micro-Raman spectrometer equipped with a He-Ne laser (λ = 633 nm) coupled with a microscope with a set of interchangeable objectives. The spectra were collected using a 100× magnification objective, and the laser intensity was varied from 0.15 to 0.75 mW. The acquisition time was varied from 5 to 10 s and the scan number from 30 to 60, depending on the sample, in order to obtain the best signal-to-noise ratio. A minimum of three spectra were collected for every sample, both on the reference gel soaked into the extraction solution (blank gel matrix) and the gel after the dyes’ extraction. All spectra were processed using OriginPro 9 software (©OriginLab): the background was subtracted fitting a polynomial baseline to the power of five, the spectra were normalised, and “adjacent averaging” smoothing was applied to reduce noise.

### 3.9. dLLME

The dLLME clean-up procedure was developed and validated to enhance dyes recovery. The development and validation study is under submission to another journal by the same authors. After the gel-supported liquid extraction from the mock-ups, the dyes were re-extracted from the gels for the HPLC-MS/MS analyses using the same aqueous solution. The gels loaded with madder, and the turmeric dyes were soaked in 0.8 mL NH_3_ (30–33%), 0.8 mL Na_2_EDTA (1 mM), and 4.4 mg NaCl. After 24 h, 495.6 mg NaCl was added together with 1 mL HCl 6 M and 0.8 mL HCOOH (≥95%) to bring the solution pH to 3. The dyes were then extracted from the aqueous phase into the organic solvent: 250 µL 2-propanol was added to every sample and, subsequently, together in the same syringe, 200 µL 1-pentanol and 100 µL 2-propanol were vigorously injected to obtain a highly dispersed thee-phasic system, known as a cloudy solution [28,45,46].

For indigo dyes, the gels were re-extracted in 1.5 mL distilled water containing NaOH:Na_2_S_2_O_4_ 1:2 (*w*/*w*). After 24 h, 530 µL HCl 6 M was added, and the solution volume was brought to 5 mL with distilled water. For the extraction into organic solvent, 750 µL 2-propanol and 100 µL chloroform were vigorously injected together to obtain the cloudy solution [46]. All samples were successively sonicated for 10 min and centrifuged for 10 min at 10,000 RPM and 5 °C. The aqueous solution was discarded. For samples containing anthraquinone dyes, 1-pentanol was washed using 3.1 mL of a solution of 2.8 M NaCl. Lastly, the organic solvent was evaporated under N_2_ flow. All samples were reconstituted with 100 µL MeOH:H_2_O 1:1 for the HPLC-MS/MS analyses.

### 3.10. HPLC-MS/MS Analyses

For the chromatographic analysis, a SCIEX Exion LC AD System was used. The system was coupled to a Sciex QTRAP 6500 mass spectrometer system with electrospray ionisation (ESI) and multicomponent IonDrive Technology.

The column chosen was a reversed phase BEH C18 (2.1 mm × 50 mm) with 1.7 µm silica particles. The injected volume was 3 µL. The mobile phases chosen were 0.1% formic acid in Millipore water (phase A) and 0.1% formic acid in acetonitrile (phase B). The gradient programme is shown in Table 4. Table 5 reports the MRM transitions used for the main target analytes’ identification (in negative) based on optimisation using certified standards. Identifications were made relying on retention times and two fragmentation transitions.

### 3.11. Colorimetric Measurements

To assess the perceptivity of the procedure on paint layers and textiles, colorimetric measurements were performed before and after gel-supported extraction, and the ΔE values were calculated. Colorimetric coordinates were acquired using fibre optic reflectance spectroscopy (FORS) in the visible range. The spectrophotometer used was a BWTEK Exemplar LS (B&W Tek, Plainsboro, NJ, USA) with a tungsten lamp BWTEK (series BPS101, 5 W, emission spectrum from 350 to 2600 nm and 2800 K colour temperature). The acquisition range was from 180 to 1100 nm, with a resolution from 0.6 nm to 6.0 nm. The fibre optic, a THORLABS RP22, was used with a head to obtain a 45° inclination of the probe. The measurements were performed in the dark, before and after gel extraction, on the same point. Every measurement was repeated three times and mediated. The CIE L*a*b* parameters were extracted from the spectra using the software BWSpec. The colour variation ΔE00 was calculated using the formula reported in the CIEDE2000 guidelines [39,50].

## 4. Conclusions

In conclusion, gel-supported in situ extraction of madder, turmeric, and indigo was applied for the first time on wool and tempera paints for SERS and HPLC-MS/MS dyes identification.

The ammonia–EDTA solution proved to be able to extract turmeric, a direct dye, from textiles. Furthermore, the solution, already tested on water soluble paint layers [36], allowed anthraquinone dye extraction also from tempera paints that were naturally aged for six months.

The dyes extracted using the ammonia–EDTA solution were identified directly on the gels after Ag colloid pastes addition by means of SERS. While madder-related peaks were clearly distinguishable on both agar gel and Nanorestore Gel^®^ HWR, no turmeric-related peaks could be detected on Nanorestore Gel^®^ HWR.

A reducing solution, already tested by Platania et al. for gel-supported extraction from cotton [16], was selected for indigoids. Nanorestore Gel^®^ HWR demonstrated to not be compatible with the reducing solution, while agar gel extraction was successful on wool and tempera paints. The SERS analysis on agar gel produced very good results, and indigoids-related peaks were clearly recognizable and highly reproducible.

A dLLME clean-up procedure was applied for the HPLC-MS/MS analyses. The procedure was necessary for the dye re-extraction from the gels and purification before injection in the instrument.

The dLLME clean-up workflow was already applied by Serafini et al. for the HPLC-MS/MS identification of anthraquinone dyes extracted from ancient textiles using the ammonia–EDTA solution. The results are under submission to another journal. The procedure demonstrated, for the first time, to be effective also for the HPLC-MS/MS identification of turmeric.

Moreover, a clean-up protocol for HPLC-MS/MS analysis of indigoids is still under development. Further dilution of the reducing solution could be effective in perfecting the dLLME procedure tested, which could not be applied because of a salt precipitation.

For both spectroscopic and chromatographic analyses, dye-related signals observed after Nanorestore Gel^®^ HWR extraction were less intense when compared to the ones observed after agar gel (3% *w*/*v*) extraction.

This is in accordance with the Nanorestore Gel^®^ HWR retention power. Hence, unless dealing with very water sensitive materials [36], agar gel use is preferable.

The colour change results on the mock-ups before and after gel-supported liquid extraction were very promising for all dyes tested. Considering the SERS and HPLC-MS/MS results, there is still a margin for improvement. The gel cylinder dimension could be consistently reduced and extraction times extended to make the procedure completely imperceptible.

Future research will be focused on artificially aged mock-ups to assess the effect of degradation phenomena on invasiveness and extraction efficiency.

Ultimately, the presented gel-supported in situ extraction is suited for multianalytical dye identification. Techniques traditionally used to study dyes, both invasively and noninvasively, can be combined without interferences from the matrix. During HPLC-MS/MS analyses, the addition of further transitions to the instrumental parameters related to the analytes of interest can allow for a more accurate portrayal of the dyes’ molecular pattern, including glycosylated moieties [28]. Thus, the approach constitutes a valid alternative to accompany noninvasive analyses of inorganic substances for comprehensive studies of cultural heritage without posing a threat to their integrity.

## Figures and Tables

**Table 1:** Main SERS scattering peaks observed in the spectra of agar gel (3% *w*/*v*) and Nanorestore Gel^®^ HWR after extraction of madder and turmeric from wool and tempera mock-ups. The vibrational modes are reported as ν (stretching) and δ (bending).

Peaks from Madder				Attribution	Peaks from Turmeric		Attribution	Peaks from the Gel Matrix	
Agar Gel (3% *w*/*v*)		Nanorestore Gel^®^ HWR			Agar Gel (3% *w*/*v*)	Nanorestore Gel^®^ HWR		Agar Gel (3% *w*/*v*)	Nanorestore Gel^®^ HWR
Tempera	Textile	Tempera	Textile		Textile	Textile		838 cm^−1^ 1000 cm^−1^ 1034 cm^−1^ 1268 cm^−1^ 1332 cm^−1^ 1383 cm^−1^ 1461 cm^−1^	839 cm^−1^ 999 cm^−1^ 1249 cm^−1^ 1321 cm^−1^ 1355 cm^−1^ 1422 cm^−1^ 1448 cm^−1^ 1760 cm^−1^
1144 cm^−1^	1155 cm^−1^	1156 cm^−1^	1160 cm^−1^	ν C-C, δ C-H	1139 cm^−1^	-	skeleton vibr.		
1270 cm^−1^	1283 cm^−1^	1290 cm^−1^	1292 cm^−1^	ν C-C, δ H-C-C	1166 cm^−1^		skeleton vibr.		
1320 cm^−1^	1321 cm^−1^	1324 cm^−1^	1315 cm^−1^	ν C-C, δ H-C-C	1290 cm^−1^		δ C-C-C, C-C-H and C=CH		
1615 cm^−1^	1614 cm^−1^	1622 cm^−1^	1611 cm^−1^	ν C=O	1600 cm^−1^		δ C-OH		

**Table 2:** Main scattering peaks observed in the spectra of agar gel 3% (*w*/*v*) after extraction of indigo from wool and tempera mock-ups. The vibrational modes are reported as ν (stretching) and δ (bending).

Peaks from Indigo		Attribution	Peaks from the Gel Matrix
Tempera	Textile		675 cm^−1^ 1000 cm^−1^ 1440 cm^−1^
544 cm^−1^	543 cm^−1^	δ C=C-CO-C and C-N	
597 cm^−1^	594 cm^−1^	δ C-C and C-N	
1224 cm^−1^	1223 cm^−1^	δ N-H and C-H	
1250 cm^−1^	1248 cm^−1^	δ C-H, C=C, and N-H	
1574 cm^−1^	1573 cm^−1^	ν C=C and C=O	

**Table 3:** Colour variation (ΔE_00_) measured for every mock-up before and after dye extraction.

Sample	Name	Description	Gel	Extraction Time	ΔE_00_
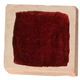	R1R	Tempera paint, madder lake pigment	Agar gel (3% *w*/*v*)	15 min	2.97
			Nanorestore Gel^®^ HWR	15 min	4.96
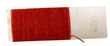	ROB	Wool dyed with madder	Agar gel (3% *w*/*v*)	3 h	2.09
			Nanorestore Gel^®^ HWR	3 h	2.76
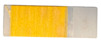	CUR	Wool dyed with turmeric	Agar gel (3% *w*/*v*)	3 h	3.87
			Nanorestore Gel^®^ HWR	3 h	5.24
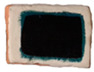	I1AR	Tempera paint, indigo over azurite	Agar gel (3% *w*/*v*)	5 min	4.14
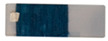	IND	Wool dyed with indigo	Agar gel (3% *w*/*v*)	5 min	1.52

**Table 4:** Chromatographic gradient used during for HPLC-MS/MS analyses.

Time (min)	Phase A: 0.1% HCOOH in H_2_O	Phase B: 0.1% HCOOH in ACN
0.00	95%	5%
1.00	95%	5%
5.00	35%	65%
5.20	0%	100%
6.50	0%	100%
7.00	95%	5%
8.50	95%	5%

**Table 5:** Instrumental parameters optimised to detect every analyte basing on MRM transitions (negative mode). The parameters were optimised relying on certified standards analysis and literature reports [47,48,49].

Analyte	Parent Ion (*m*/*z*)	Fragments (*m*/*z*)	DP (V)	EP (V)	CE (V)	CXP (V)
Alizarin	239	210	−120	−8.1	−49	−15
		167			−40	−10
Purpurin	255	227	−109	−8.3	−38	−21
		171			−42	−13
Curcumin	367	134	−40	−2.5	−48	−13
		158			−44	−14

## Data Availability

Data will be made available on request.
